# Effiziente Therapie des leichten Morbus Crohn und der leichten Colitis ulcerosa

**DOI:** 10.1007/s00108-024-01840-x

**Published:** 2024-12-23

**Authors:** Gerhard Rogler

**Affiliations:** Klinik für Gastroenterologie und Hepatologie, Universitätsspital Zürich, Rämistrasse 100, 8091 Zürich, Schweiz

**Keywords:** Chronisch-entzündliche Darmerkrankungen, Topische Therapie, Orale Therapie, 5‑Aminosalicylate, Budesonid, Inflammatory bowel disease, Administration, topical, Administration, oral, 5‑Aminosalicylic acid, Budesonide

## Abstract

Grundstein der Behandlung der leichten Colitis ulcerosa ist nach wie vor die perorale oder topische (rektale) Anwendung von 5‑Aminosalicylaten (5-ASA). Häufig werden bei der leichten Colitis ulcerosa 5‑ASA-Präparate nur peroral verabreicht. Dabei zeigen Studiendaten, dass bei der Proctitis ulcerosa und der linksseitigen Kolitis rektale 5‑ASA-Applikationen sogar wirksamer sind als die orale Verabreichung. In einem nächsten Schritt sollten steroidhaltige topische Therapien eingesetzt werden. Beim leichten Morbus Crohn kommen primär topische Steroide wie Budesonid zum Einsatz. Bei symptomfreien Patienten ist aber umstritten, ob eine Therapie notwendig ist. Es fehlt nach wie vor die Evidenz, die nachweisen würde, dass die aggressivere Behandlung (mit Immunsuppressiva, Biologika oder „small molecules“) bei diesen Patienten einen Vorteil für den Langzeitverlauf hat. In den meisten Leitlinien wird der Einsatz von 5‑ASA bei leichtem Morbus Crohn kritisch gesehen. Dennoch existiert eine gewisse Evidenz für eine ausreichend hoch dosierte Therapie mit 5‑ASA, wobei man sich der limitierten Wirksamkeit bewusst sein muss. Für den postoperativen Einsatz von 5‑ASA bei einem leichten Rezidiv gibt es dagegen klare Evidenz.

## Abstract (en)

The cornerstone of treatment for mild ulcerative colitis is still the oral or topical (rectal) application of aminosalicylates (5-ASA). 5‑ASA preparations are often only administered orally in mild ulcerative colitis. Study data show that in ulcerative proctitis and left-sided colitis, rectal 5‑ASA preparations are even more effective than oral administration. In a next step, steroid-containing topical therapies should be used. Topical steroids such as budesonide are also primarily used in mild Crohn’s disease. However, it is controversial whether treatment is necessary in symptom-free patients. There is still a lack of evidence to prove that more aggressive treatment (using immunosuppressants, biologics or small molecules) has a long-term benefit in these patients. Most guidelines are critical of the use of 5‑ASA in mild Crohn’s disease. Nevertheless, there is some evidence for sufficiently high-dose treatment with 5‑ASA, although one must be aware of its limited effectiveness. However, there is clear evidence for the postoperative use of 5‑ASA in cases of mild recurrence.

Die Definition des leichten Morbus Crohn oder der leichten Colitis ulcerosa bzw. die Abgrenzung von schwereren Formen ist umstritten. In unterschiedlichen klinischen Therapiestudien, aber auch in epidemiologischen Arbeiten wurden unterschiedliche Indizes zur Abgrenzung von mittelschweren oder schweren Verlaufsformen verwendet. Solche Aktivitätsindizes können in Einzelfällen klinische Hilfsmittel zur Aktivitätsbeurteilung chronisch-entzündlicher Darmerkrankungen (CED) darstellen [34]. Viele der Indizes, die zur Aktivitätsbeurteilung von CED herangezogen werden, sind jedoch recht kompliziert und werden daher häufig nur in klinischen Studien genutzt, um den Effekt einer therapeutischen Intervention möglichst klar quantifizieren zu können. Trotz allem besteht meist durchaus eine Übereinkunft darüber, ob eine CED eher leicht oder schon mittelschwer ist. Um die Schwere der CED einzuschätzen, reichen im Allgemeinen klinische Symptome sowie Calprotectin als Biomarker und gegebenenfalls eine Bildgebung mittels Ultraschall oder Endoskopie [34].

Entgegen dem, was man auf dem Boden aktueller Diskussionen über das „window of opportunity“ sowie über das Hit-hard-and-early- oder Top-down-Konzept erwarten könnte, zeigt ein signifikanter Anteil der Patienten mit CED eine leichte Verlaufsform [4]. Die Studienevidenz für die Behandlung dieser Patienten ist lückenhaft, in Bezug auf Biologika oder „small molecules“ fehlt sie sogar gänzlich, da meist mindestens eine mittelschwere oder schwere Erkrankung vorhanden sein muss, damit Patienten in eine klinische (Zulassungs‑)Studie für neue Medikamente eingeschlossen werden können [16]. Im Falle der Colitis ulcerosa hat – zumindest zu Beginn der Erkrankung – mehr als die Hälfte der Patienten eine leichte Aktivität [3]. Die Behandlung insbesondere des leichten Morbus Crohn, aber auch der leichten Colitis ulcerosa ist eine der wichtigsten offenen Fragen in diesem Feld.

## Therapie des leichten Morbus Crohn

Bei einem leichten Morbus Crohn ohne signifikante klinische Symptome stellt sich die Grundsatzfrage, ob überhaupt eine Therapie notwendig ist. Immer wieder wird eine Ileitis zufällig bei einer (Vorsorge‑)Koloskopie oder bei einer Ultraschalluntersuchung aus anderen Gründen entdeckt. Unklar ist dabei immer, wann der Beginn der Ileitis war, da sie ja symptomlos ist. Würde man den Hit-hard-and-early- oder den Window-of-opportunity-Postulaten folgen, müsste man in dieser Situation eine Behandlung empfehlen. Aber auch mit den momentan effektivsten Medikamenten wird eine mukosale Heilung nur bei etwa einem Drittel der Patienten erreicht, wobei diese Zahlen aus Studien mit Patienten stammen, die eine moderate bis schwere Entzündung aufweisen. Daher ist die Evidenz für eine Behandlung in einer solchen Situation sehr schwach. Es gibt in einer solchen Situation keine klinischen Kriterien für den Behandlungserfolg. Aus den genannten Gründen erscheint es völlig vertretbar, bei symptomlosen Patienten mit leichter Ileitis nicht zu behandeln. Hier ist selbstverständlich zu berücksichtigen, wie der/die Betroffene zu einer Behandlung eingestellt ist. Ohne dies durch entsprechende Studien belegen zu können, erscheinen beispielsweise sonographische Follow-ups sinnvoll, um frühzeitig mögliche Komplikationen wie Strikturen erkennen zu können.

Als Erstlinientherapie bei leichtem Morbus Crohn ist eine Gabe von 5‑Aminosalicylaten (5-ASA) möglich. Aktuelle Leitlinien, wie die Leitlinien der European Crohn’s and Colitis Organisation (ECCO), sprechen sich gegen die Anwendung von 5‑ASA bei leichtem Morbus Crohn aus [10]: „5-Aminosalicylate spielt in der modernen Behandlung von Morbus Crohn, unabhängig von der Krankheitslokalisation, keine Rolle, da es durchgehend keine Belege für ihre Wirksamkeit gibt“ [10]. Hierzu seien folgende Anmerkungen erlaubt: Als Bestätigung wird eine Cochrane-Metaanalyse angeführt, die im Gegensatz zur plakativen Aussage der Leitlinie zu einem differenzierteren Bild kommt [21]. In diese Metaanalyse wurden zehn Studien eingeschlossen. Die Autoren fanden einen (nichtsignifikanten) Trend zugunsten von Sulfasalazin gegenüber Placebo bei der Remissionsinduktion, wobei der Nutzen hauptsächlich auf Patienten mit Crohn-Kolitis beschränkt war [21]. Ein Anteil von 45 % der Patienten mit Sulfasalazin erreichte nach 17–18 Wochen eine Remission, verglichen mit 29 % der Patienten mit Placebo. Eine Remissionsrate von 45 % ist sicher nicht überragend, aber auch nicht desaströs. Es gab keinen Unterschied zwischen Sulfasalazin und Placebo in Bezug auf die Nebenwirkungen, sodass die Therapie als sehr sicher angesehen werden kann. Sulfasalazin war deutlich weniger wirksam als Steroide (60 % mit Remission nach 17–18 Wochen). Allerdings zeigten die mit Sulfasalazin behandelten Patienten signifikant weniger Nebenwirkungen als die Patienten, die Kortikosteroide erhielten (relatives Risiko [RR] 0,43; [21]).

In der Erstlinientherapie bei leichtem Morbus Crohn ist die Gabe von 5‑Aminosalicylaten möglich

Eine Netzwerkmetaanalyse aus dem Jahr 2017 fand einen kleinen, aber statistisch signifikanten Behandlungsvorteil für 5‑ASA gegenüber Placebo hinsichtlich der Remissionsinduktion bei Patienten mit Morbus Crohn, wenn Dosen > 2,4 g/Tag verwendet wurden [5]. Eine weitere Netzwerkmetaanalyse war jedoch negativ [24]. Eine gepoolte Analyse von drei doppelt verblindeten, placebokontrollierten Studien mit einem langsam freisetzenden 5‑ASA-Präparat in einer Dosierung von 4 g fand eine signifikant bessere Verringerung des Crohn’s Disease Activity Index (CDAI) im Vergleich zu Placebo [15]. Die absolute Verringerung des CDAI war jedoch niedrig. Eine Cochrane-Analyse von Therapien zur postoperativen Rezidivprophylaxe bei Patienten mit Morbus Crohn kommt zu dem Schluss, dass Adalimumab (Hazard Ratio [HR] 0,11, 95 %-Konfidenzintervall [KI] 0,02–0,33; „Evidenz mit niedriger Sicherheit“) und 5‑ASA (HR 0,69, 95 %-KI 0,53–0,87; „Evidenz mit *mittlerer* Sicherheit“) die Wahrscheinlichkeit eines klinischen Rezidivs im Vergleich zu Placebo verringern können [17].

Wie können nun diese teilweise widersprüchlichen Befunde interpretiert werden? Ist 5‑ASA oder Sulfasalazin bei leichtem Morbus Crohn wirksam? Im Gegensatz zu den ECCO-Leitlinien kann man die vorliegenden Daten auch dahingehend interpretieren, dass ein Therapieversuch sinnvoll sein kann – dies allerdings nur, wenn die Morbus-Crohn-Läsionen oberflächlich sind und die Dosierung ausreichend hoch gewählt wird (3 g und mehr). 5‑ASA wird von intestinalen Epithelzellen rasch resorbiert und durch das Enzym N‑Acetyltransferase im Darmepithel und der Leber umfassend zu N‑Acetyl-5-ASA acetyliert [7, 32, 33]. Klinische Studien deuten auf eine erhebliche interindividuelle Variabilität in Bezug auf den raschen Abbau von 5‑ASA hin [28]. Wenn bei schnellerer Acetylierung kein intaktes und damit wirksames 5‑ASA mehr in tieferen Darmwandschichten ankommt, kann es auch keine antientzündlichen Effekte haben [28]. Hingegen kann 5‑ASA bei auf die Mukosa beschränkten Läsionen ebenso wie bei der Colitis ulcerosa wirksam sein. Leider gibt es derzeit keinerlei klinische Standardverfahren, mit denen ermittelt werden könnte, ob bei einem Patienten eine schnelle oder langsame Acetylierung gegeben ist. Daher kann man einen Therapieversuch mit ausreichend dosiertem 5‑ASA bei leichtem Morbus Crohn mit oberflächlichen Läsionen (typischen Aphthen) unternehmen, muss aber bereit sein, bei mangelnder Wirkung, die Therapie rasch zu eskalieren.

Die nächste Therapiestufe besteht in der Applikation topisch wirksamer Steroide wie Budesonid. In den aktuellen ECCO-Leitlinien (Empfehlung 1.2) wird empfohlen, Budesonid zur Remissionsinduktion bei Patienten mit aktivem leichtem bis mittelschwerem Morbus Crohn zu verwenden, wenn die Entzündung auf das Ileum und/oder Colon ascendens beschränkt ist [10]. Entsprechende Präparationen sind für Morbus Crohn zugelassen. Allerdings gibt es natürlich auch Präparationen, die höhere Konzentrationen im linksseitigen Kolon erreichen, für die Colitis ulcerosa zugelassen sind und durchaus „off label“ hier eingesetzt werden können. Eine systematische Cochrane-Metaanalyse fasste drei Studien mit einer Dosierung von 9 mg Budesonid/Tag zusammen: Budesonid war Placebo hinsichtlich des Erreichens eines klinischen Ansprechens und einer klinischen Remission (RR 1,93) bei Patienten mit leicht aktivem Morbus Crohn im Dünn- und/oder Dickdarm überlegen [19, 31]. Im Vergleich zu herkömmlichen Steroiden, wie Prednisolon oder Methylprednisolon, die normalerweise mit vielen systemischen Nebenwirkungen verbunden sind, zeigte Budesonid eine gute topische entzündungshemmende Wirkung, jedoch nur eine geringe systemische Bioverfügbarkeit und daher ein deutlich besseres Sicherheitsprofil [31].

Mit 6 mg Budesonid kann über einen gewissen Zeitraum einem erneuten Schub vorgebeugt werden

Welche Strategien sollte man zur Remissionserhaltung verfolgen, wenn mit Budesonid eine Remission erreicht werden konnte? Der Versuch einer Remissionserhaltung mit 3‑mal 1 mg Budesonid täglich war hinsichtlich der Rezidivraten Placebo nicht überlegen [11, 12]. In einer Metaanalyse kamen Sandborn u. Hanauer zu dem Schluss, dass die Dosis von 6 mg Budesonid/Tag bei Patienten mit leichtem Morbus Crohn und medikamentös induzierter Remission die Zeit bis zu einem Rezidiv verlängern kann und die Rezidivrate nach 3 und 6 Monaten (jedoch nicht nach 12 Monaten) signifikant senkt [13, 36]. Mit 6 mg Budesonid kann also über einen gewissen Zeitraum einem erneuten Schub vorgebeugt werden. Allerdings bewirken auch 6 mg gerade bei älteren Patienten vermehrt Steroidnebenwirkungen, sodass der Einsatz über längere Zeiträume wie beispielsweise die oben erwähnten 6 Monate sorgfältig abgewogen werden sollte.

**Abb. 1:**
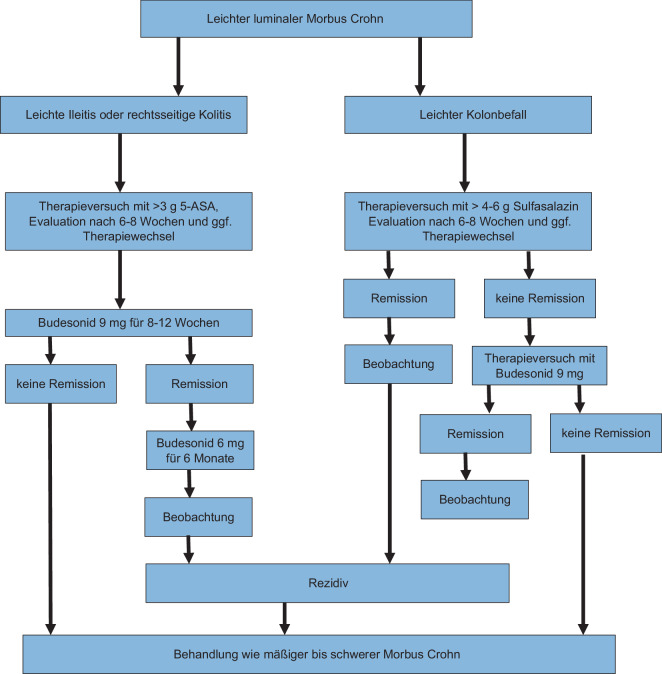
Therapiealgorithmus für die Behandlung des leichten Morbus Crohn. *5‑ASA* 5‑Aminosalicylate. (Modifiziert nach [39])

Bei einem Versagen dieser Ansätze sollte der leichte Morbus Crohn wie eine mittelschwere oder schwere Erkrankung behandelt werden (Abb. 1).

## Therapie der leichten Colitis ulcerosa

**Abb. 2:**
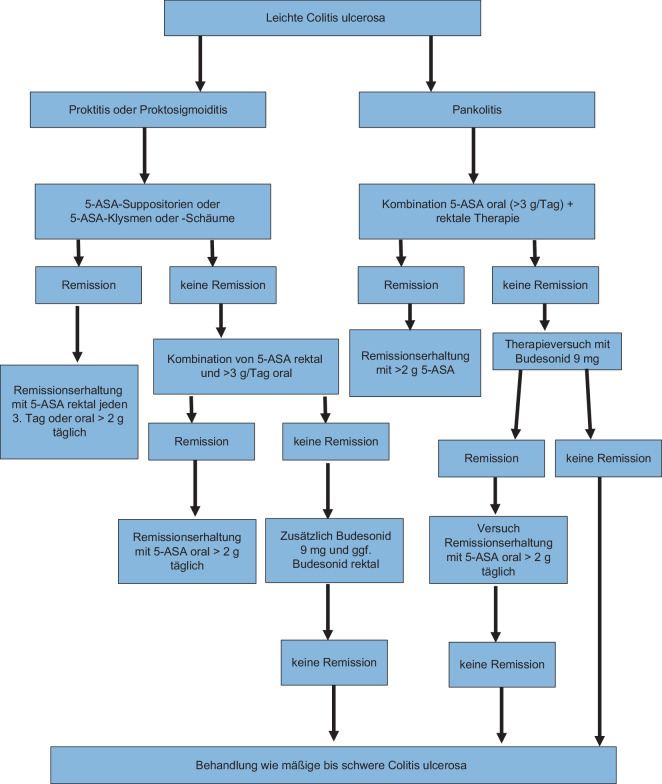
Therapiealgorithmus für die leichte Colitis ulcerosa. *5‑ASA* 5‑Aminosalicylate

Bei der Colitis ulcerosa erscheint auch im Falle einer leichten Ausprägung eine Therapie sinnvoll (Abb. 2). Sie reduziert das Risiko eines kolitisassoziierten Karzinoms. Zudem kann eine mukosale Heilung bei der Colitis ulcerosa mit einer deutlich höheren Wahrscheinlichkeit als beim Morbus Crohn erreicht und damit die Langzeitprognose verbessert werden. Da eine Colitis ulcerosa normalerweise im Rektum am schwersten ausgeprägt ist, ist eine Symptomfreiheit auch bei leichter Entzündung wesentlich seltener. Die Anwendung von 5‑ASA kann weiterhin als die Erstlinientherapie und Standardbehandlung zur Einleitung einer Remission bei leichter Colitis ulcerosa angesehen werden, insbesondere wenn die Patienten bisher noch keine Therapie erhalten haben [20, 30, 38]. Dies wurde in zahlreichen Metaanalysen (unter anderem auch in einer Cochrane-Metaanalyse [25]) bestätigt [1, 8, 26]. Es besteht zudem eine klare Evidenz, dass die Kombination von oraler und rektaler 5‑ASA-Applikation der alleinigen oralen Therapie deutlich überlegen ist [1, 29]. Eine Studie von Safdi et al. [35] zeigte dies 1997 sehr eindrücklich: Bereits nach 3 Wochen führte die Kombinationstherapie zu einer deutlich besseren Reduktion der Entzündung als die alleinige Behandlung mit 5‑ASA-Einläufen oder 5‑ASA-Tabletten. Im Vergleich zu Patienten, die 5‑ASA-Einläufe oder 5‑ASA-Tabletten erhielten, berichteten Patienten mit der Kombinationstherapie signifikant früher über das Fehlen von Blut im Stuhl [35]. Ford et al. [8] zeigten in einer Metaanalyse, dass die intermittierende topische (rektale) 5‑ASA-Behandlung der kontinuierlichen oralen 5‑ASA-Therapie hinsichtlich der Remissionserhaltung überlegen ist.

Warum ist dies so? Für 5‑ASA existiert eine klare Dosis-Wirkungs-Beziehung. Je höher die lokalen Konzentrationen an der Darmschleimhaut sind, umso besser ist die Wirkung [9]. Daher ergibt die Kombination von rektaler und oraler Therapie auch sehr viel Sinn. Dadurch können insbesondere im Rektum und Sigma an der Mukosa besonders hohe Konzentrationen erreicht werden. Auch die Einnahme einer oralen Therapie mit 4,8 g 5‑ASA/Tag zeigt bessere Ergebnisse als eine Therapie mit 2,4 g [14].

Mit 5‑ASA wird so bei einer signifikanten Anzahl von Patienten eine mukosale Heilung erzielt [18, 22]. Kann durch 5‑ASA eine mukosale Heilung erreicht werden, schützt dies vor Entzündungsschüben [23]. Die kumulative Rezidivrate nach einem Jahr betrug 23 % bei Patienten mit klinischer und endoskopischer Remission und 80 % bei Patienten mit ausschließlich klinischer Remission [23]. Höhere Dosen erzielen dabei wiederum höhere Raten an mukosaler Heilung [27].

Zur Remissionseinleitung sollten laut Therapieleitlinien orales und rektales 5‑ASA kombiniert werden

Daher empfehlen die gültigen Therapieleitlinien für die leichte Colitis ulcerosa zur Einleitung einer Remission eine Kombination aus oralem 5‑ASA (2,0–4,8 g/Tag) und rektalem 5‑ASA (Zäpfchen 1 g/Tag bei Proktitis und Einlauf ≥ 1 g/Tag bei linksseitiger Kolitis/Pankolitis) über 8 Wochen [6, 30].

Leider wird diese Empfehlung nur sehr eingeschränkt umgesetzt [37]. In einer Untersuchung der Swiss IBD Cohort Study erhielten nur 26 % der Patienten mit Proktitis eine rektale Therapie mit 5‑ASA und 13 % eine kombinierte systemische und rektale Behandlung, während 29 % eine systemische Behandlung mit 5‑ASA ohne rektale Behandlung erhielten [37]. Es ist offensichtlich, dass mit diesem Ansatz nicht die optimalen Wirkstoffkonzentrationen erreicht werden können. Der Anteil der rektalen Arzneimittelanwendung sank von 39 % bei Proktitis auf 13,1 % bei Pankolitis trotz der klaren Empfehlung, auch bei leichter Pankolitis eine rektale Therapie einzusetzen [37]. Bei fehlendem Ansprechen auf orales 5‑ASA sollte daher zunächst daran gedacht werden, Dosis und Verabreichungsweg zu optimieren. Nach rektaler 5‑ASA-Applikation sollte der Patient auf dem Bauch oder auf der linken Seite liegen. Das Präparat sollte für mindestens 20–30 min im Intestinaltrakt verbleiben. Dies kann (bei leichter Erkrankung) durch Gabe von 1 bis 2 Tabletten Loperamid 20–30 min vor 5‑ASA-Applikation unterstützt werden.

5‑ASA ist ein sehr sicheres Präparat und Nebenwirkungen sind sehr selten. Einmal jährlich sollte die sehr seltene interstitielle Nephritis (1:2000 bis 1:5000) durch eine Untersuchung des Urinsediments ausgeschlossen werden. Eine Messung des Kreatinins ist zur Überwachung der Nierenfunktion weniger geeignet, da sich eine Kreatininerhöhung erst nach erheblicher und möglicherweise irreversibler Schädigung der Niere einstellt.

Wird mit 5‑ASA – in optimaler Dosierung und Applikationsform – kein ausreichender Therapieerfolg erzielt, sollte im nächsten Schritt Budesonid eingesetzt werden. In einer Metaanalyse von 15 Studien mit 4083 Teilnehmern wurden Budesonid-Multimatrix (MMX) 9 mg/Tag und 5‑ASA > 2,4 g/Tag verglichen. Sie zeigten eine ähnliche Wirksamkeit für das Erreichen einer klinischen und endoskopischen Remission und waren beide Placebo deutlich überlegen [2]. Bei linksseitiger, distaler Kolitis kann auch Budesonidschaum erfolgreich eingesetzt werden, bei Proktitis Budesonidsuppositorien [40]. Auch bei Colitis ulcerosa gilt, dass der Langzeiteinsatz von Budesonid 9 mg über einen Zeitraum von 8 bis 12 Wochen hinaus zu Steroidnebenwirkungen führen kann, insbesondere bei älteren Patienten. Eine steroidabhängige Situation (Wiederauftreten von Symptomen nach Absetzen oder Reduktion von Budesonid) erfordert immer eine Therapieanpassung mit Immunsuppression, Biologika oder „small molecules“. Das Gleiche gilt, wenn die leichte Colitis ulcerosa mit 5‑ASA oder Budesonid nicht erfolgreich behandelt werden kann. Die Behandlungsschwelle ist dabei individuell. Bei leichten Symptomen und weiter bestehender Kolitis sowie mangelndem Therapieansprechen können entsprechend den Leitlinien beispielsweise Vedolizumab oder auch Sphingosin-1-Phosphat-Rezeptor-Modulatoren (Ozanimod, Etrasimod) verabreicht werden. Nutzen und Behandlungsrisiken sind hier sorgfältig abzuwägen. Wird eine Remission der Colitis ulcerosa erreicht, kann statt 5‑ASA zur Remissionserhaltung auch *Escherichia* *coli* Stamm Nissle (Mutaflor®) eingesetzt werden. Das Präparat hat sich als gleichwertig („non-inferior“) erwiesen.

Diäten haben sich bei der Colitis ulcerosa nicht als wirksam erwiesen. Sicherlich kann aber eine gesunde Lebensweise zu einem insgesamt günstigen Verlauf beitragen.

## Fazit für die Praxis

- Bei leichter Colitis ulcerosa ist eine Therapie grundsätzlich empfohlen (auch zur Malignomprävention), während die Therapieindikation bei Morbus Crohn von Beschwerden, Befunden und Patientenpräferenz abhängig ist.
- 5‑Aminosalicylate (5-ASA) in rektaler und oraler Form ist das Medikament der Wahl zur Behandlung einer leichten Colitis ulcerosa. Die rektale 5‑ASA-Anwendung ist die wirksamste Applikationsart und kann durch orale Gabe unterstützt werden.
- Die Gabe von 5‑ASA wird bei leichtem Morbus Crohn nicht von den Leitlinien unterstützt, ein Therapieversuch ist vor allem bei oberflächlichem Befall und Crohn-Kolitis sinnvoll.
- Die Gabe lokal wirksamer Steroide (Budesonid) ist bei leichter chronisch-entzündlicher Darmerkrankung (CED) ebenfalls kurzzeitig wirksam, wobei die Effizienz bei Langzeitgabe abnimmt.
- Spricht eine leichte CED nicht auf die vorhandenen Therapieoptionen an, sollte sie wie eine mittelschwere bis schwere Erkrankung behandelt werden.
- Leichte CED wurden bisher nur unzureichend untersucht, und die Wirksamkeit moderner Biologika und „small molecules“ ist unbekannt. Diese Wissenslücke sollte durch weitere Studien geschlossen werden.

## References

[1] Barberio B, Segal JP, Quraishi MN et al (2021) Efficacy of Oral, Topical, or Combined Oral and Topical 5‑Aminosalicylates, in Ulcerative Colitis: Systematic Review and Network Meta-analysis. J Crohns Colitis 15:1184–1196 33433562 10.1093/ecco-jcc/jjab010

[2] Bonovas S, Nikolopoulos GK, Piovani D et al (2019) Comparative assessment of budesonide-MMX and mesalamine in active, mild-to-moderate ulcerative colitis: A systematic review and network meta-analysis. Br J Clin Pharmacol 85:2244–2254 31269287 10.1111/bcp.14051 PMC6783624

[3] Burisch J, Katsanos KH, Christodoulou DK et al (2019) Natural disease course of ulcerative colitis during the first five years of follow-up in a European population-based inception cohort-an Epi-IBD study. J Crohns Colitis 13:198–208 30289522 10.1093/ecco-jcc/jjy154

[4] Burisch J, Kiudelis G, Kupcinskas L et al (2019) Natural disease course of Crohn’s disease during the first 5 years after diagnosis in a European population-based inception cohort: an Epi-IBD study. Gut 68:423–433 29363534 10.1136/gutjnl-2017-315568

[5] Coward S, Kuenzig ME, Hazlewood G et al (2017) Comparative Effectiveness of Mesalamine, Sulfasalazine, Corticosteroids, and Budesonide for the Induction of Remission in Crohn’s Disease: A Bayesian Network Meta-analysis: Republished. Inflamm Bowel Dis 23:E26–E37 30052985 10.1097/MIB.0000000000001158

[6] D’Amico F, Magro F, Dignass A et al (2024) Practical management of mild-to-moderate ulcerative colitis: an international expert consensus. Expert Rev Gastroenterol Hepatol 18:421–430 39225555 10.1080/17474124.2024.2397650

[7] Fischer C, Maier K, Stumpf E et al (1983) Disposition of 5‑aminosalicylic acid, the active metabolite of sulphasalazine, in man. Eur J Clin Pharmacol 25:511–515 6140167 10.1007/BF00542120

[8] Ford AC, Khan KJ, Achkar JP, Moayyedi P (2012) Efficacy of oral vs. topical, or combined oral and topical 5‑aminosalicylates, in Ulcerative Colitis: systematic review and meta-analysis. Am J Gastroenterol 107:167–176 22108446 10.1038/ajg.2011.410

[9] Frieri G, Giacomelli R, Pimpo M et al (2000) Mucosal 5‑aminosalicylic acid concentration inversely correlates with severity of colonic inflammation in patients with ulcerative colitis. Gut 47:410–414 10940280 10.1136/gut.47.3.410 PMC1728031

[10] Gordon H, Minozzi S, Kopylov U et al (2024) ECCO Guidelines on Therapeutics in Crohn’s Disease: Medical Treatment. J Crohns Colitis 10.1093/ecco-jcc/jjae091 38877997

[11] Gross V (2008) Oral pH-modified release budesonide for treatment of inflammatory bowel disease, collagenous and lymphocytic colitis. Pharmacother, Bd. 9. Expert, Opin, S 1257–1265 10.1517/14656566.9.7.1257 18422482

[12] Gross V, Andus T, Ecker KW et al (1998) Low dose oral pH modified release budesonide for maintenance of steroid induced remission in Crohn’s disease. The Budesonide Study Group. Gut 42:493–496 9616309 10.1136/gut.42.4.493 PMC1727061

[13] Hanauer S, Sandborn WJ, Persson A, Persson T (2005) Budesonide as maintenance treatment in Crohn’s disease: a placebo-controlled trial. Aliment Pharmacol Ther 21:363–371 15709986 10.1111/j.1365-2036.2005.02338.x

[14] Hanauer SB, Sandborn WJ, Kornbluth A et al (2005) Delayed-release oral mesalamine at 4.8 g/day (800 mg tablet) for the treatment of moderately active ulcerative colitis: the ASCEND II trial. Am J Gastroenterol 100:2478–2485 16279903 10.1111/j.1572-0241.2005.00248.x

[15] Hanauer SB, Stromberg U (2004) Oral Pentasa in the treatment of active Crohn’s disease: A meta-analysis of double-blind, placebo-controlled trials. Clin Gastroenterol Hepatol 2:379–388 15118975 10.1016/s1542-3565(04)00122-3

[16] Hanzel J, Ma C, Jairath V, Group IBDTD (2022) Design of Clinical Trials for Mild to Moderate Crohn’s Disease. Gastroenterology 162:1800–1814 35240140 10.1053/j.gastro.2022.02.036

[17] Iheozor-Ejiofor Z, Gordon M, Clegg A et al (2019) Interventions for maintenance of surgically induced remission in Crohn’s disease: a network meta-analysis. Cochrane Database Syst Rev 9:CD13210 31513295 10.1002/14651858.CD013210.pub2 PMC6741529

[18] Kamm MA, Sandborn WJ, Gassull M et al (2007) Once-daily, high-concentration MMX mesalamine in active ulcerative colitis. Gastroenterology 132:66–75 17241860 10.1053/j.gastro.2006.10.011

[19] Kuenzig ME, Rezaie A, Kaplan GG et al (2018) Budesonide for the Induction and Maintenance of Remission in Crohn’s Disease: Systematic Review and Meta-Analysis for the Cochrane Collaboration. J Can Assoc Gastroenterol 1:159–173 30656288 10.1093/jcag/gwy018 PMC6328928

[20] Lamb CA, Kennedy NA, Raine T et al (2019) British society of gastroenterology consensus guidelines on the management of inflammatory bowel disease in adults. Gut 68:s1–s106 31562236 10.1136/gutjnl-2019-318484 PMC6872448

[21] Lim WC, Wang Y, MacDonald JK, Hanauer S (2016) Aminosalicylates for induction of remission or response in Crohn’s disease. Cochrane Database Syst Rev 7:CD8870 27372735 10.1002/14651858.CD008870.pub2 PMC6457996

[22] Marshall JK, Irvine EJ (2000) Putting rectal 5‑aminosalicylic acid in its place: the role in distal ulcerative colitis. Am J Gastroenterol 95:1628–1636 10925961 10.1111/j.1572-0241.2000.02180.x

[23] Meucci G, Fasoli R, Saibeni S et al (2012) Prognostic significance of endoscopic remission in patients with active ulcerative colitis treated with oral and topical mesalazine: a prospective, multicenter study. Inflamm Bowel Dis 18:1006–1010 21830282 10.1002/ibd.21838

[24] Moja L, Danese S, Fiorino G et al (2015) Systematic review with network meta-analysis: comparative efficacy and safety of budesonide and mesalazine (mesalamine) for Crohn’s disease. Aliment Pharmacol Ther 41:1055–1065 25864873 10.1111/apt.13190

[25] Murray A, Nguyen TM, Parker CE et al (2020) Oral 5‑aminosalicylic acid for induction of remission in ulcerative colitis. Cochrane Database Syst Rev 8:CD543 32786164 10.1002/14651858.CD000543.pub5 PMC8189994

[26] Nguyen NH, Fumery M, Dulai PS et al (2018) Comparative efficacy and tolerability of pharmacological agents for management of mild to moderate ulcerative colitis: a systematic review and network meta-analyses. Lancet Gastroenterol Hepatol 3:742–753 30122356 10.1016/S2468-1253(18)30231-0 PMC6821871

[27] Papi C, Fasci-Spurio F, Rogai F et al (2013) Mucosal healing in inflammatory bowel disease: treatment efficacy and predictive factors. Dig Liver Dis 45:978–985 24018244 10.1016/j.dld.2013.07.006

[28] Perrotta C, Pellegrino P, Moroni E et al (2015) Five-aminosalicylic Acid: an update for the reappraisal of an old drug. Gastroenterol Res Pract 2015:456895 25685145 10.1155/2015/456895 PMC4320793

[29] Probert CS, Dignass AU, Lindgren S et al (2014) Combined oral and rectal mesalazine for the treatment of mild-to-moderately active ulcerative colitis: rapid symptom resolution and improvements in quality of life. J Crohns Colitis 8:200–207 24012063 10.1016/j.crohns.2013.08.007

[30] Raine T, Bonovas S, Burisch J et al (2022) ECCO Guidelines on Therapeutics in Ulcerative Colitis: Medical Treatment. J Crohns Colitis 16:2–17 34635919 10.1093/ecco-jcc/jjab178

[31] Rezaie A, Kuenzig ME, Benchimol EI et al (2015) Budesonide for induction of remission in Crohn’s disease. Cochrane Database Syst Rev 2015:CD296 26039678 10.1002/14651858.CD000296.pub4 PMC10613338

[32] Rijk MC, van Hogezand RA, van Schaik A, van Tongeren JH (1989) Disposition of 5‑aminosalicylic acid from 5‑aminosalicylic acid-delivering drugs during accelerated intestinal transit in healthy volunteers. Scand J Gastroenterol 24:1179–1185 2574905 10.3109/00365528909090784

[33] Rijk MC, van Schaik A, van Tongeren JH (1988) Disposition of 5‑aminosalicylic acid by 5‑aminosalicylic acid-delivering compounds. Scand J Gastroenterol 23:107–112 2894070 10.3109/00365528809093858

[34] Rogler G, Biedermann L (2020) Klassifikationen, Indizes, Aktivitätsbeurteilung. In: Hoffmann JC et al (Hrsg) Chronisch-entzündliche Darmerkrankungen. Springer, Heidelberg

[35] Safdi M, DeMicco M, Sninsky C et al (1997) A double-blind comparison of oral versus rectal mesalamine versus combination therapy in the treatment of distal ulcerative colitis. Am J Gastroenterol 92:1867–1871 9382054

[36] Sandborn WJ, Lofberg R, Feagan BG et al (2005) Budesonide for maintenance of remission in patients with Crohn’s disease in medically induced remission: a predetermined pooled analysis of four randomized, double-blind, placebo-controlled trials. Am J Gastroenterol 100:1780–1787 16086715 10.1111/j.1572-0241.2005.41992.x

[37] Seibold F, Fournier N, Beglinger C et al (2014) Topical therapy is underused in patients with ulcerative colitis. J Crohns Colitis 8:56–63 23566922 10.1016/j.crohns.2013.03.005

[38] Singh S, Feuerstein JD, Binion DG, Tremaine WJ (2019) AGA Technical Review on the Management of Mild-to-Moderate Ulcerative Colitis. Gastroenterology 156:769–808 30576642 10.1053/j.gastro.2018.12.008 PMC6858923

[39] Wong K, Bressler B (2008) Mild to moderate Crohn’s disease: an evidence-based treatment algorithm. Drugs 68:2419–2425 19016571 10.2165/0003495-200868170-00002

[40] Zeng J, Lv L, Mei ZC (2017) Budesonide foam for mild to moderate distal ulcerative colitis: A systematic review and meta-analysis. J Gastroenterol Hepatol 32:558–566 27699863 10.1111/jgh.13604

